# Cognitive Profile Discrepancy as a Possible Predictor of Emotion Dysregulation in a Clinical Sample of Female Adolescents with Suicidal Behavior

**DOI:** 10.3390/ejihpe14120202

**Published:** 2024-12-19

**Authors:** Flora Furente, Federica Annecchini, Emilia Matera, Sabrina Serafino, Giorgia Frigeri, Alessandra Gabellone, Lucia Margari, Maria Giuseppina Petruzzelli

**Affiliations:** 1 Department of Translational Biomedicine and Neuroscience (DIBRAIN), University of Studies of Bari “Aldo Moro”, 70124 Bari, Italy; flora.furente@uniba.it (F.F.); serafino.sabrina@gmail.com (S.S.); giorgia.fri@icloud.com (G.F.); alessandra.gabellone@uniba.it (A.G.); maria.petruzzelli@uniba.it (M.G.P.); 2 Department of Precision and Regenerative Medicine and Ionian Area (DIMEPRE-J), University of Studies of Bari “Aldo Moro”, 70124 Bari, Italy; emilia.matera@uniba.it (E.M.); lucia.margari@uniba.it (L.M.)

**Keywords:** adolescents, emotion dysregulation, self-harm, suicide, Wechsler scales, cognitive profile, IQ, general ability index, cognitive proficiency index

## Abstract

Emotional dysregulation (ED) has not yet been defined as a clinical entity, although it plays an important role in child and adolescent psychopathology. It is a transdiagnostic construct defined as the inability to regulate the intensity and quality of emotions to produce an appropriate emotional response, to cope with excitability, mood instability, and emotional over-reactivity. The aim of this study is to assess, in a sample of female patients with internalizing disorders and suicidal behavior, the correlation between cognitive profile (assessed with Wechsler Scales) and the dimensions of emotion regulation assessed with the Difficulties in Emotion Regulation Scale (DERS). We also investigated whether a discrepancy between the General Ability Index (GAI) and the Cognitive Proficiency Index (CPI) could have predictive value for certain ED domains. Our results confirmed a statistically significant prediction of the ΔGAI-CPI for individual DERS domains and for the total (*p* = 0.014 for DERS-TOT, *p* = 0.04 for GOALS, *p* = 0.002 for STRATEGIES and *p* = 0.015 for CLARITY); furthermore, IAG and PRI correlate with worse ability to find ER strategies (*p* = 0.04, *p* = 0.010). These results suggest the importance of examining the impact of cognitive vulnerabilities on the ability to manage emotions and psychopathology in general, even with normal FSIQ/GAI.

## 1. Introduction

Individuals react to emotional responses by using strategies that can flexibly modify emotional experience. This is referred to as emotional regulation (ER), which is the ability to recognize, evaluate, modify, and manage emotions in personal and socially acceptable ways to cope with strong feelings and maintain appropriate adaptability [1]. ER is considered a multidimensional construct that includes willingness to experience negative or positive emotions; awareness, understanding, and acceptance of different emotional states; commitment to achieving a specific goal in response to positive and negative emotions; flexible use of appropriate strategies to modulate the intensity and/or duration of the emotional response; and displacement, not repression, of the dysfunctional emotion [2].

Emotion dysregulation (ED) refers to a maladaptive or aberrant ER process defined as the inability to regulate the intensity and quality of emotions in order to produce an appropriate emotional response, to cope with excitability, mood instability, and emotional over-reactivity, and to return to an emotional baseline [3]. This construct is widely used in both clinical and not-clinical groups from different cultural contexts and ages. Indeed, it is a universal dimension that can play a key role in interpersonal [4,5,6,7] and intrapersonal [8,9,10,11] variables.

Although, in clinical settings, the concept of ED is historically linked to the scientific research on borderline personality disorder, deficits in emotion management are now considered in the literature as a transdiagnostic dimension in various domains of psychopathology, also for children and adolescents, including symptoms of increased reactivity and quickness to angry, anxious, or depressed affect [12,13]. In addition ED has been shown to be a risk factor for various forms of suicidal behavior including suicidal ideation (SI), suicidal plans (SP), suicide attempts (SA), and non-suicidal self-injury (NSSI) in adolescents who use these as maladaptive ER strategies against overwhelming intense negative emotions [3,14,15]. Various pathways for the relationship between ED and suicide risk have been proposed in the literature, such as the interpersonal theory of suicide (IPTS), in which emotion dysregulation has been consistently linked to interpersonal difficulties [16,17] and the Integrated Motivational–Volitional Model of Suicidal Behavior (IMV) which describes stages leading to suicidal behavior [18]. Other studies examined other markers of suicide risk such as electrodermal hypo-reactivity [19,20,21].

Understanding the key determinants of suicide risk is an important concern, especially in recent decades when more than 700,000 people end their lives each year [22]. In addition, epidemiologic studies have shown that suicidality is a major problem in early adults and in adolescence: young people aged 18–30 years have the highest prevalence of reported suicidal ideation and are more likely to plan suicide than older adults [23,24], and among adolescents, suicide is the second leading cause of death among youth aged 10–19 years in the United States [25]. In addition to age, female gender has also been identified as a risk factor for suicidal behavior [26]. Thus, internalizing disorders, including depression and anxiety, as well as psychological distress and suicidal behavior, have been shown to increase significantly in the adolescent population in many countries, with the female gender being more affected [27]. Gender-specific differences in mental disorders are well known in the literature. For example, females are about twice as likely to suffer from mood and anxiety disorders, while males are about four times more likely to suffer from impulsive disorders [28].

Adolescence is characterized by marked functional and structural changes at multiple levels, including the neural circuits underlying emotional processes. Changes in the temporal dynamics of emotional processes (e.g., arousal tone, peak duration, and width of tolerance window) occur during this developmental period and have been associated with risk for mood and anxiety disorders, borderline personality disorder, post-traumatic stress disorder and bipolar disorder [29,30,31,32,33,34,35,36,37], suggesting a complex interaction between biological and environmental factors.

Among the six basic strategies of ER [38], the ability to cognitively reappraise events by interpreting them in ways that change emotional responses to them [39,40] is so flexible and effective, that it is an important component of cognitive behavioral therapies for various disorders. Reappraisal depends on well-studied complex cognitive abilities such as executive functions (EFs) including working memory, attention, and response selection; it appears that reappraisal is particularly linked to working memory [41,42,43].

The cognitive strategies that adolescents use to cope with negative emotions might have different profiles that could be associated with different psychopathological symptoms, including suicidality. While emotions in childhood are mainly regulated on an external and behavioral level (e.g., through parental support and crying), ER becomes more internal and cognitive in adolescence.

To our knowledge, there are conflicting data on the association between ER deficit and suicidality [44], especially when comparing studies based on adults and adolescents [45]. Moreover, few authors correlate ER dimensions with the cognitive functioning profile, as the two have largely been studied separately. In line with observations in clinical practice, this study aims to investigate the hypothesis that poorer coping skills with negative emotions in adolescent girls with internalizing disorders and suicidal behavior are related to discrepancies in cognitive profile and greater difficulties in executive functions.

Therefore, understanding the nature of the relationship between ED and cognitive profiles could have implications for more targeted approaches to risk assessment and intervention. So, the study is focused on these objectives:

(1) the cognitive profiles and ED dimensions in a clinical sample of female adolescents with internalizing disorders and suicidal behavior;
(2) the correlation between cognitive profiles and ED dimensions;
(3) the predictive value of cognitive profiles for certain ED dimensions.

## 2. Materials and Methods

### 2.1. Participants

The study included a gender-homogeneous (female) group of patients aged between 12 and 18 years belonging to middle, and high school, referred to the Department of Child and Adolescent Neuropsychiatry, Translational Biomedicine and Neurosciences (DiBraiN) Department, of the University of Bari, Italy, from January 2018 to January 2023. Patients were included in the study if they met the criteria for a diagnosis of an internalizing disorder, according to the revised 5th edition of the Diagnostic and Statistical Manual of Mental Disorders Text Revision (DSM-5-TR) [46] and suicidal behavior. A formal diagnosis of Intellectual Developmental Disorder, autism spectrum disorder, attention-deficit/hyperactivity disorder, Schizophrenic Spectrum Disorders—which are often characterized by an in-homogeneous cognitive profile—and failure to complete the protocols required for the study were considered exclusion criteria for participation in the study. All participants underwent a global clinical assessment that included the current psychopathological symptoms evaluated using the Youth Self-Report (YSR) questionnaire [47]; neuropsychiatric diagnosis formulated according to DSM-5-TR criteria [46]; and the history of neurodevelopmental and psychopathological symptoms and the presence of suicidal behavior including NSSI, SI, and SA, both through an anamnestic survey by neuropsychiatrists. Cognitive profiles and ED were assessed psychometrically. The study was approved by the Ethics Committee of Bari (study number 6888, prot. 0063972 18 July 2022).

### 2.2. Measurements

#### 2.2.1. Cognitive Performance

Patients underwent an assessment of cognitive abilities using the Wechsler Intelligence Scale for Children (WISC-IV) [48] if they were less than 16 years and 11 months old, while the Wechsler Adult Intelligence Scale (WAIS-IV) was used if they were older than this [49]. The first instrument showed a reliability of 0.75 to 0.90 in the Cornoldi research (2013) using Cronbach’s alpha method [50]; the second instrument’s estimates of internal consistency again showed that Cronbach’s alpha was very high (0.91) for the WAIS-IV scale and for four-factor structures (between 0.81 and 0.95) [51]. Wechsler Scales are clinical instruments that assess an Intelligence Quotient (Full Scale IQ or FSIQ) and four cognitive domains using different cognitive indices: Verbal Comprehension Index (VCI), Perceptual Reasoning Index (RPI), Working Memory Index (WMI) and Processing Speed Index (PSI). Composite scores can also be calculated as an option. The General Ability Index (GAI) is recommended if a person shows significant discrepancies between the indices. It is derived from the results of the verbal comprehension and perceptual reasoning subtests and provides an estimate of general intellectual ability. The Cognitive Proficiency Index (CPI) is a measure of efficient and competent information processing and is derived from the working memory and processing speed tasks. The CPI is the counterpart to the GAI and theoretically represents a person’s cognitive processing competence [52]. Efficient cognitive processing frees-up cognitive resources for more complex or higher-level tasks. For this study, the arithmetic difference between the GAI and CPI (ΔGAI-CPI) was also calculated. This is an effective parameter to investigate discrepancies between the WISC and WAIS indices. Indeed, results in the literature have shown that GAI-CPI or GAI-FSIQ discrepancies are an effective criterion for discriminating between groups and that they have been well studied in specific learning disabilities (SLDs) but are still understudied in psychopathology [53].

#### 2.2.2. ER

The psychometric assessment of ER was performed using the Difficulties in Emotion Regulation Scale (DERS), a 36-item self-report questionnaire, validated in Italy for use with adolescents that assesses six relevant domains of ER [54]. The total scores obtained in each of the subtests were recorded, including (1) non-acceptance of emotional responses (NON ACCEPTANCE—6 items), (2) difficulties in goal-directed behavior (GOALS—5 items), (3) difficulties with impulse control (IMPULSE—8 items), (4) lack of emotional awareness (AWARENESS—5 items), (5) limited access to emotion regulation strategies (STRATEGIES—6 items) and (6) lack of emotional clarity (CLARITY—5 items). The sum of the subscale scores determines the total score (DERS-TOT), with high scores corresponding to greater difficulties in emotion regulation and showing strong internal consistency, test–retest reliability and good validity as well as a Cronbach’s alpha of 0.90 for the total scale, and values between 0.74 and 0.88 for the six subscales [55,56].

### 2.3. Statistical Analyses

All variables were recorded in structured forms developed specifically for this study. The analyses were performed using the open-source software JASP (0.19.1 version for Apple Silicon) [57]. Qualitative data on the diagnosis and clinical characteristics of the sample are expressed by numbers and frequencies; quantitative data such as age and the scores of the psychometric questionnaires and Wechsler scales are expressed by means, medians, and standard deviations (SD). Shapiro’s test was used to assess their normal distribution. The relationship between WISC-IV/WAIS-IV indices and ED was examined using Spearman’s correlation. Linear regressions were performed to assess the predictive power of the cognitive indices on DERS domains. The significance level was set at a *p*-value < 0.05.

## 3. Results

The sample comprised 74 female patients with a mean age of 15.1 years (+1.5 sd). The sample met DSM-5-TR diagnostic criteria for depressive disorders, anxiety disorders, feeding and eating disorders, adjustment disorders, and somatic symptom and related disorders. Suicidal behaviors occurred mainly as NSSI. The sample scored higher on the internalizing-problems scale and internalizing disorders DSM-oriented scales of the YSR questionnaire. This sample’s demographic and clinical characteristics are shown in Table 1.

The Wreshler indices and DERS scores of the sample are summarized in Table 2. The arithmetic difference between GAI and CPI (ΔGAI-CPI) is shown in the last column. The *p*-value of the Shapiro–Wilk test for normal distribution shows that all of the DERS scores are not normally distributed, with the exception of awareness.

For this reason, Spearman’s correlation was calculated to estimate the relationship between the Wreshler indices and the DERS scores. The results are shown in Table 3. Statistically significant correlations were found between ΔGAI-CPI and DERS-TOT (*p* = 0.010), GOALS (*p* = 0.010), STRATEGIES (*p* = 0.002), and CLARITY (*p* = 0.017); there is also a statistically significant correlation between STRATEGIES and PRI (*p* = 0.04) as well as GAI (*p* = 0.010). There are directly proportional correlations between ΔGAI-CPI and DERS-TOT, GOALS, STRATEGIES and CLARITY. In addition, IAG and PRI correlate with a poorer ability to find ER strategies.

Lastly, four separate linear regression models were run with ΔGAI-CPI as the independent variable, with each of the DERS scores showing a significant correlation with it. All covariates showed a significant predictive role of ΔGAI-CPI with the following *p*-values: *p* = 0.014 for DERS-TOT, *p* = 0.04 for GOALS, *p* = 0.002 for STRATEGIES and *p* = 0.015 for CLARITY. Thus, using a linear regression model, we verified that a larger discrepancy between GAI and CPI can be considered predictive (variable independent) for the dimensions of ER that appear correlated. In addition, two linear regression models were run to examine the predictive role of GAI and PRI for STRATEGIES. The regression models are summarized in Table 4. Each regression model was controlled for AGE, not changing the significance (Table S5 in Supplementary Materials).

**Figure 1:**
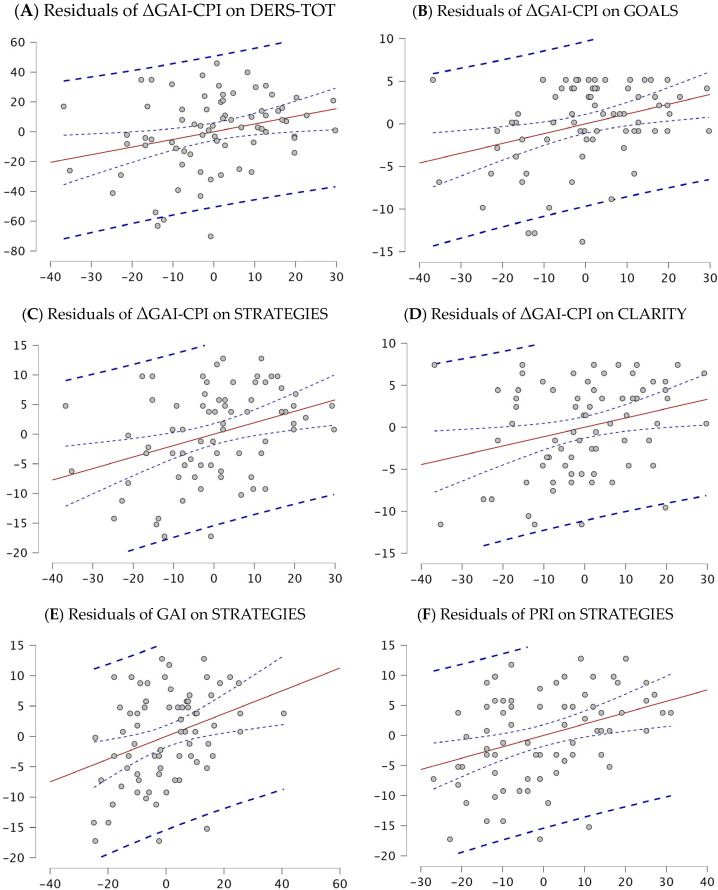
(**A**–**F**) Partial residual plots of each regression model; the residuals are the vertical distances between the test set data points and the model’s red regression line, blue lines represent their distribution and shape.

The direct proportional increase in ED parameters with higher discrepancies in ΔGAI-CPI are shown in the partial residual plots reported in Figure 1A–D. Figure 1E,F show residuals of GAI and PRI on STRATEGIES.

## 4. Discussion

The main finding of this study was that a cognitive profile characterized by lower WMI and PSI scores compared to VCI and PRI could predict a higher ED total score in a sample of adolescent female patients diagnosed with internalizing disorders and a clinical history of suicidal behavior. The same relationship exists between WMI and PSI and the DERS domains related to difficulty engaging in goal-directed behaviors, managing strategies of ER, and having clarity in ER. In addition, higher GAI and PRI scores could predict a poorer ability to find ER strategies.

Several studies and systematic reviews have already examined the associations between suicide and specific emotional difficulties and maladaptive strategies in their regulation [58,59,60,61,62,63]; a recent systematic review by Rogante et al. (2024), focusing on adults, found significant positive associations between ED and SI, with less evidence for SA [64]. Another study that focused on children and adolescents showed that affective and behavioral dysregulation were related to suicidal ideation both acutely (over weeks) and chronically (over months). This suggests that suicidal behavior and suicidal attempts are similar to self-injurious behavior in the fact that they may be attempts to reduce intolerable emotional states and have reinforcing properties [65].

More recently, studies have been conducted on the extent to which a person’s specific cognitive profile can help with neurological and neurodevelopmental diagnosis [53,66]. This is consistent with the “Specificity” theory, according to which the pattern of strengths and weaknesses (PSW) within the neuropsychological functioning, rather than generalized cognitive problems, may lead to a more targeted approach to specific diagnosis (the theory is mainly studied in specific learning disabilities) [67,68]. To our knowledge, this approach has never been used in the study of psychopathological problems.

Past literature has described how higher IQ, particularly FSIQ, has been associated with an increased risk of NSSI in female adolescents [45]. This contrasts with findings reported in studies based on adults wherein lower FSIQ was a risk factor for suicide, particularly in males [69,70,71]. There are conflicting data on the association between EF deficits and suicidality [44]. However, it can be speculated that people self-harm to cope with “emotional cascades” [72], resulting from the rumination of negative emotions until the emotional stimuli intensify and become a burden. Thus, the suppression of negative emotions or failure to use adaptive cognitive strategies impedes effective emotion regulation and individuals engage extreme behaviors, such as NSSI, to reduce these aversive emotions [73]. There is consistent literature on the bi-directional link between ER abilities and EF [40,43]. Indeed, EF impairment affects the ability to process and interpret information to respond to situational demands and can be influenced by the patient’s emotional state, altering their performance in cases of high psychological distress [74]. Other studies confirmed the role of EF in the ability to control ER. Indeed, individual differences in working memory capacity predict success in the voluntary regulation of emotional expression and emotional experience [43]. In addition, cognitive alterations may also increase difficulties in interpersonal relationships, which are a classic trigger for suicidal behavior [75]. A recent study by Kim et al. (2024) showed that the best predictor of lifetime NSSI engagement was rumination; specifically, high levels of rumination were associated with lower levels of working memory, and higher risk of NSSI [76]. Our results can be considered consistent with the findings of Kim et al. Namely, female adolescents with poorer working memory abilities and processing speed might display a ruminative attitude because they are unable to use their working memory capacity for another task to distract themselves from the negative stimuli. In particular, female adolescent patients with internalizing disorders were recruited in our sample if they had currently or in the past shown self-injurious behavior. The sample showed an average level of FSIQ and its cognitive domains and a high level of ED scores. The correlation matrix between the cognitive profile and DERS dimensions shows that the only cognitive parameter related to DERS-TOT score is the value resulting from the difference between GAI and CPI (ΔGAI-CPI). Furthermore, no proportional correlation between the GAI/FSIQ or the CPI and DERS-TOT of the emotion dysregulation index was found in our study. This underscores the importance of the inhomogeneity of the cognitive profile with lower performance in EF for the relationship with higher ED scores, which is a transdiagnostic dimension of internalizing psychopathology associated with self-injurious behavior.

## 5. Limitations

The study also has severe limitations that reduce the significance of the results. First of all, the sample size is small, which reduces the statistical power of the study and does not allow the research to be enriched by stratifying the sample, especially given the fact that it is difficult to find the same clinical phenotype in male adolescents in clinical practice. However, it is known that ER and the underlying biological and environmental processes differ according to sex and age, so the results obtained are valid when applied to a sex-homogeneous sample. In addition, greater scientific rigor could have been achieved by better understanding the impact of psychiatric comorbidities and controlling for their severity or stratifying the sample by principal diagnosis. Moreover, the lack of detailed sociodemographic information on economic and cultural status does not allow the use of control variables other than age. The assessment of suicidal behavior using only an anamnestic survey and without questionnaires and scales reduces the scientific rigor of the statistical analysis, and this is another limitation of the study. Therefore, the results should be interpreted with caution until replicated in larger and more diverse samples.

## 6. Conclusions, Clinical Implications, and Future Perspective

The high levels of ED exhibited by female adolescents with suicidal behavior and internalizing symptoms are related to cognitive profiles characterized by high discrepancies between verbal/perceptual intelligence and lower processing abilities. Since weaknesses in the subclinical neuropsychological profiles could better highlight the worst psychopathological outcomes of these patients, the use of complete and multiparametric intelligence scales should be a basic rule in the clinical assessment of patients with mental health problems.

This study is based on the clinical evidence that a specific cognitive profile with weaknesses in working memory and processing speed is frequently found in female adolescent patients when assessed for internalizing disorders and suicidality. A better understanding of this relationship will allow clinicians to prevent risky behaviors in mental health problems through earlier interventions. Thus, these data suggest that there is a need to further investigate the relationship between specific cognitive profiles and the transdiagnostic dimension of ER to better understand the impact of cognitive vulnerabilities, even in the presence of a normal FSIQ/GAI, on the ability to manage emotions and psychopathology in general. In fact, the aim of this research was to put neuropsychology at the forefront of guiding clinical practice from the diagnostic to the therapeutic approach, not only in neurodevelopmental disorders such as specific and non-specific learning disabilities, autism spectrum disorder, and attention deficit/hyperactivity disorder, but also in the assessment of clinical phenotypes in psychopathology. This could be an interesting starting point for future research, to increase the sample size and better assess the psychiatric comorbidities, in order to develop a tailored diagnostic and therapeutic approach in adolescent mental health.

## Figures and Tables

**Table 1:** The demographic and clinical features of the sample.

Diagnosis		*n* (%)
	Depressive Disorders	51 (68.9%)
	Anxiety Disorders	33 (44.6%)
	Feeding and Eating Disorders	23 (31.1%)
	Adjustment Disorders	12 (16.2%)
	Somatic Symptom and Related Disorders	10 (13.6%)
**Suicidal/Self-harming behaviors**		***n* (%)**
	NSSI	52 (70.3%)
	SI	30 (40.5%)
	SA	12 (16.2%)
**Age**		**m (sd)**
		15.1 (1.5)
**YSR scales**		
	Total Problems	67.6 (11.2)
	Internalizing Problems	71.1 (13.4)
	Externalizing Problems	58.3 (12.1)
	DSM—Affective Problems	73.3 (12.2)
	DSM—Anxiety Problems	66.0 (9.2)
	DSM—Somatic Problems	63.9 (11.5)

**Table 2:** A descriptive table of the sample by Weschler indices and DERS scores.

Wreshler Indices								
	FSQI	VCI	PRI	WML	PSI	GAI	CPI	ΔGAI-CPI
**N**	74							
**Mean**	105	104	112	95.2	99.4	108	97.3	10.8
**SD**	14.8	15.9	14.2	16.1	15.2	13.4	12.8	13.9
**Shapiro p**	0.45	0.453	0.062	0.161	0.313	0.485	0.095	0.763
**DERS Scores**								
	**Ders-Tot**	**Non Accept**	**Goals**	**Impulse**	**Awareness**	**Strategies**	**Clarity**	
**N**	74							
**Mean**	119	17.8	19.8	19.1	18.9	26.2	17.6	
**SD**	25	6.9	4.78	7.32	5.25	7.63	5.5	
**Shapiro p**	0.023	0.017	<0.001	0.044	0.135	0.039	0.001	

**Table 3:** Correlation matrix with Spearman’s indices between Wechsler indices and DERS scores.

Spearman’s Correlations								
Variable		VCI	PRI	WML	PSI	GAI	CPI	ΔGAI-CPI
**Ders-Tot**	*Spearman’s rho*		0.145			0.150		0.298
	*p*-*value*		0.217			0.203		0.010 *
**Goals**	*Spearman’s rho*		0.062			0.066		0.298
	*p*-*value*		0.601			0.578		0.010 *
**Strategies**	*Spearman’s rho*		0.327			0.299		0.361
	*p*-*value*		0.004 *			0.010 *		0.002 *
**Clarity**	*Spearman’s rho*		0.115			0.150		0.277
	*p*-*value*		0.328			0.203		0.017 *

* Only intersections with significant correlations are reported.

**Table 4:** (A–F). Linear regression models with their significances.

	Independent Variable	Covariate	*p*-Value
A.	ΔGAI-CPI	DERS-TOT	0.014
B.		DERS Goals	0.004
C.		DERS Strategies	0.002
D.		DERS Clarity	0.015
E.	GAI	DERS Strategies	0.004
F.	RPI	DERS Strategies	0.002

## Data Availability

The data presented in this study are available on request from the corresponding author.

## Supplementary Materials

The following supporting information can be downloaded at: https://www.mdpi.com/article/10.3390/ejihpe14120202/s1, Table S1: Demographic and clinical features of the sample, Table S2: Descriptive table of the sample by Weschler indices and DERS scores, Table S3: Correlation matrix with Spearman’s indices between Wechsler indices and DERS scores, Table S4: (A–F). Linear regression models Table S5: (A–F). Linear regression models with AGE as controlled variable.
