# Supplementary material for: Cognitive Profile Discrepancy as a Possible Predictor of Emotion Dysregulation in a Clinical Sample of Female Adolescents with Suicidal Behavior
Source: Eur J Investig Health Psychol Educ. 2024 Dec 19;14(12):3087–98. doi: 10.3390/ejihpe14120202 (PMC11675136; doi:10.3390/ejihpe14120202)
Supplement: Supplementary file 1 [file ejihpe-14-00202-s001.zip › ejihpe-3237027-supplementary.pdf]

Table S1. Demographic and clinical features of the sample.

**Diagnosis**		**n (%)**
	Depressive Disorders	51 (68,9%)
	Anxiety Disorders	33 (44,6%)
	Feeding and Eating Disorders	23 (31,1%)
	Adjustment Disorders	12 (16,2%)
	Somatic Symptom and Related Disorders	10 (13,6%)
**Suicidal/Self-harming behaviors**		**n (%)**
	NSSI	52 (70,3%)
	SI	30 (40,5%)
	SA	12 (16,2%)
		**m (sd)**
**Age**		15,1 (1,5)
**YSR scales**		
	Total Problems	67,6 (11,2)
	Internalizing Problems	71,1 (13,4)
	Externalizing Problems	58,3 (12,1)
	DSM - Affective Problems	73,3 (12,2)
	DSM - Anxiety Problems	66,0 (9,2)
	DSM - Somatic Problems	63,9 (11,5)

Table S2. Descriptive table of the sample by Weschler indices and DERS scores.

	**Wreshler Indices**							
	**FSQI**	**VCI**	**PRI**	**WML**	**PSI**	**GAI**	**CPI**	**ΔGAI-CPI**
**N**	74							
**Mean**	105	104	112	95,2	99.4	108	97.3	10.8
**SD**	14.8	15.9	14.2	16.1	15.2	13.4	12.8	13.9
**Shapiro p**	0.45	0.453	0.062	0.161	0.313	0.485	0.095	0.763

	**DERS Scores**						
	**DERS-TOT**	**NON ACCEPT**	**GOALS**	**IMPULSE**	**AWARENESS**	**STRATEGIES**	**CLARITY**
**N**	74						
**Mean**	119	17.8	19.8	19.1	18.9	26.2	17.6
**SD**	25	6.9	4.78	7.32	5.25	7.63	5.5
**Shapiro p**	0.023	0.017	<0.001	0.044	0.135	0.039	0.001

Table S3. Correlation (complete) matrix with Spearman's indices between Wechsler indices and DERS scores.

Spearman's Correlations									
**Variable**		**FSIQ**	**VCI**	**PRI**	**WML**	**PSI**	**GAI**	**CPI**	**ΔGAI-CPI**
**DERS-TOT**	*Spearman's rho*	0.045	0.107	0.145	0.027	-0.153	0.150	-0.091	0.298
	p-value	0.704	0.365	0.217	0.817	0.192	0.203	0.441	0.010*
**NON ACCEPT**	*Spearman's rho*	0.053	0.070	0.104	0.026	-0.088	0.119	-0.063	0.212
	p-value	0.655	0.556	0.379	0.825	0.457	0.314	0.596	0.070
**GOALS**	*Spearman's rho*	-0.048	0.036	0.062	-0.067	-0.195	0.066	-0.197	0.298
	p-value	0.683	0.759	0.601	0.571	0.097	0.578	0.093	0.010*
**IMPULSE**	*Spearman's rho*	-0.058	-0.021	-0.088	0.055	-0.021	-0.066	-0.017	-0.014
	p-value	0.624	0.859	0.458	0.640	0.860	0.575	0.887	0.907
**AWARENESS**	*Spearman's rho*	9.875×10^{-4}	7.431×10^{-4}	0.088	0.018	-0.040	0.050	0.025	0.052
	p-value	0.993	0.995	0.458	0.881	0.736	0.673	0.833	0.661
**STRATEGIES**	*Spearman's rho*	0.218	0.199	0.327	0.105	-0.069	0.299	0.016	0.361
	p-value	0.062	0.089	0.004*	0.374	0.558	0.010*	0.894	0.002*
**CLARITY**	*Spearman's rho*	0.075	0.147	0.115	0.026	-0.149	0.150	-0.067	0.277
	p-value	0.528	0.211	0.328	0.826	0.205	0.203	0.572	0.017*

Table S4. (A-F). Linear regression models.

A.

Model Coefficient DERS TOT

					95% CI	
	**Unstandardized**	**Standard Error**	**t**	**p**	**Lower**	**Upper**
(Intercept)	113.596	3.557	31.932	< .001	106.504	120.688
DELTA IAG-ICC	0.514	0.203	2.529	0.014	0.109	0.919

B.

Model Coefficient DERS goals

					95% CI	
	**Unstandardized**	**Standard Error**	**t**	**p**	**Lower**	**Upper**
(Intercept)	18.602	0.669	27.823	< .001	17.269	19.935
DELTA IAG-ICC	0.115	0.038	3.010	0.004	0.039	0.191

C.

Model Coefficient DERS Strategies

					95% CI	
	**Unstandardized**	**Standard Error**	**t**	**p**	**Lower**	**Upper**
(Intercept)	24.156	1.059	22.804	< .001	22.045	26.268
DELTA IAG-ICC	0.193	0.060	3.187	0.002	0.072	0.313

D.

Model Coefficient DERS Clarity

					95% CI	
	**Unstandardized**	**Standard Error**	**t**	**p**	**Lower**	**Upper**
(Intercept)	16.372	0.782	20.925	< .001	14.812	17.931
DELTA IAG-ICC	0.111	0.045	2.489	0.015	0.022	0.200

E.

Model Coefficient DERS Strategies

					95% CI	
	**Unstandardized**	**Standard Error**	**t**	**p**	**Lower**	**Upper**
(Intercept)	5.999	6.811	0.881	0.381	-7.580	19.577
IAG	0.188	0.063	2.993	0.004	0.063	0.312

F.

Model Coefficient DERS Strategies

					95% CI	
	**Unstandardized**	**Standard Error**	**t**	**p**	**Lower**	**Upper**
(Intercept)	5.082	6.676	0.761	0.449	-8.226	18.390
IRP	0.189	0.059	3.193	0.002	0.071	0.307

Table S5. (A-F). Regression models with AGE as controlled variable

							95% CI	
Model		Unstandardized	Standard Error	Standardized	t	p	Lower	Upper
M_0	(Intercept)	119.122	2.910		40.942	< .001	113.323	124.920
M_1	(Intercept)	113.596	3.557		31.932	< .001	106.504	120.688
	DELTA IAG–ICC	0.514	0.203	0.286	2.529	0.014	0.109	0.919

Note. The following covariate was considered but not included: AGE.

Coefficients ▼

							95% CI	
Model		Unstandardized	Standard Error	Standardized	t	p	Lower	Upper
M_0	(Intercept)	19.838	0.556		35.678	< .001	18.730	20.946
M_1	(Intercept)	7.827	5.142		1.522	0.132	−2.426	18.079
	DELTA IAG–ICC	0.116	0.037	0.338	3.117	0.003	0.042	0.191
	AGE	0.715	0.338	0.229	2.113	0.038	0.040	1.389

Coefficients ▼

							95% CI	
Model		Unstandardized	Standard Error	Standardized	t	p	Lower	Upper
M_0	(Intercept)	26.230	0.887		29.575	< .001	24.462	27.997
M_1	(Intercept)	12.544	8.283		1.514	0.134	−3.972	29.060
	DELTA IAG–ICC	0.194	0.060	0.354	3.233	0.002	0.074	0.314
	AGE	0.770	0.545	0.155	1.413	0.162	−0.316	1.857

Coefficients ▼

Model		Unstandardized	Standard Error	Standardized	t	p	95% CI Lower	95% CI Upper
M_0	(Intercept)	17.568	0.639		27.490	< .001	16.294	18.841
M_1	(Intercept)	9.285	6.145		1.511	0.135	−2.968	21.538
	DELTA IAG-ICC	0.112	0.045	0.284	2.515	0.014	0.023	0.201
	AGE	0.470	0.404	0.131	1.163	0.249	−0.336	1.276

Coefficients

Model		Unstandardized	Standard Error	Standardized	t	p	95% CI Lower	95% CI Upper
M_0	(Intercept)	26.230	0.887		29.575	< .001	24.462	27.997
M_1	(Intercept)	−10.587	11.205		−0.945	0.348	−32.929	11.755
	IAG	0.210	0.062	0.369	3.364	0.001	0.085	0.334
	AGE	0.940	0.545	0.189	1.723	0.089	−0.148	2.027

Coefficients

Model		Unstandardized	Standard Error	Standardized	t	p	95% CI Lower	95% CI Upper
M_0	(Intercept)	26.230	0.887		29.575	< .001	24.462	27.997
M_1	(Intercept)	−10.587	11.205		−0.945	0.348	−32.929	11.755
	IAG	0.210	0.062	0.369	3.364	0.001	0.085	0.334
	AGE	0.940	0.545	0.189	1.723	0.089	−0.148	2.027

Coefficients ▼

Model		Unstandardized	Standard Error	Standardized	t	p
M_0	(Intercept)	111.878	1.652		67.706	< .001
M_1	(Intercept)	108.675	15.791		6.882	< .001
	DERS strategies	0.686	0.208	0.368	3.296	0.002
	AGE	−0.982	1.034	−0.106	−0.949	0.346
